# Supplementary figures and images for: Comparative analysis of Hymenasplenium (Aspleniaceae) chloroplast genomes from China
Source: PeerJ. 2024 Dec 18;12:e18667. doi: 10.7717/peerj.18667 (PMC11662895; doi:10.7717/peerj.18667)

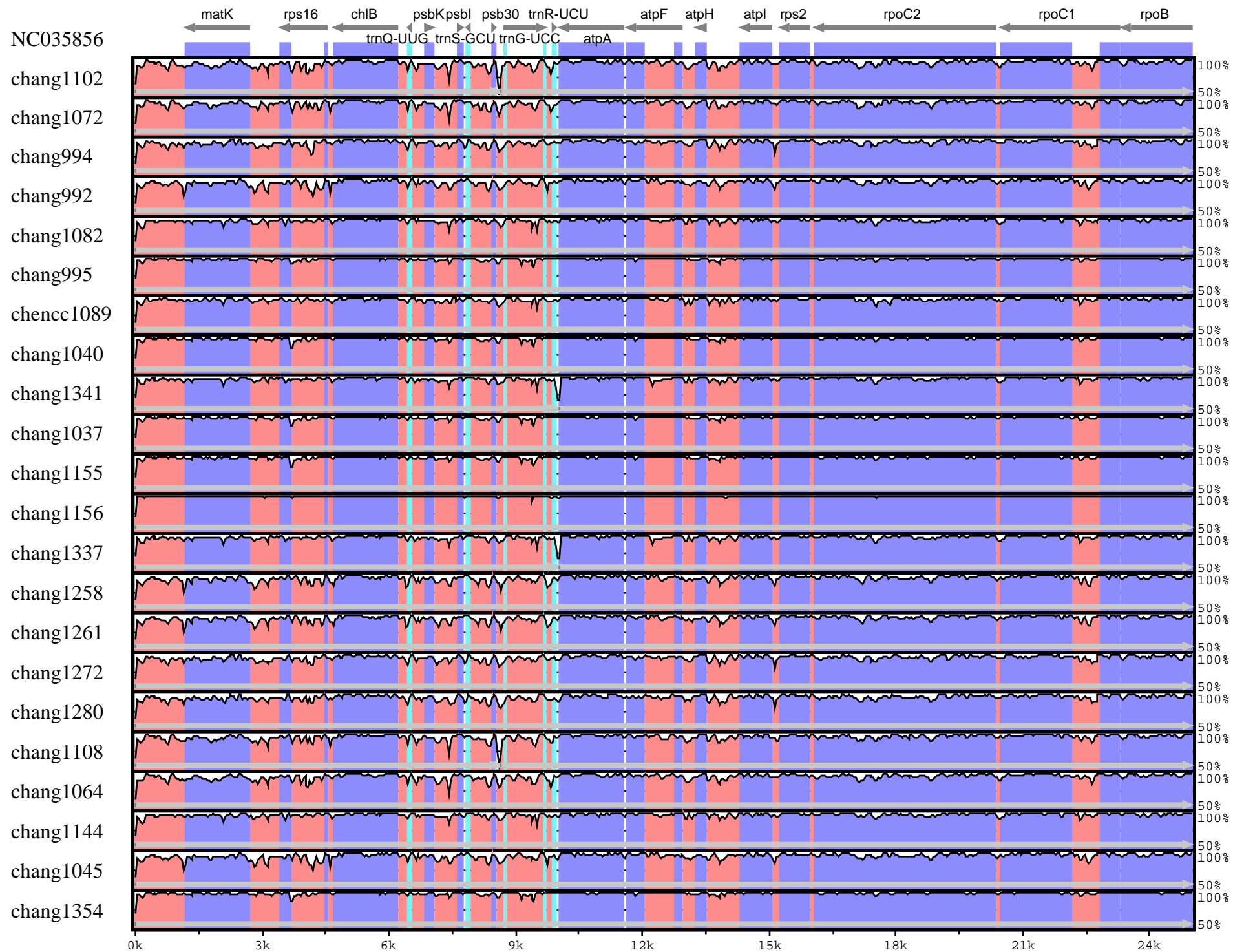

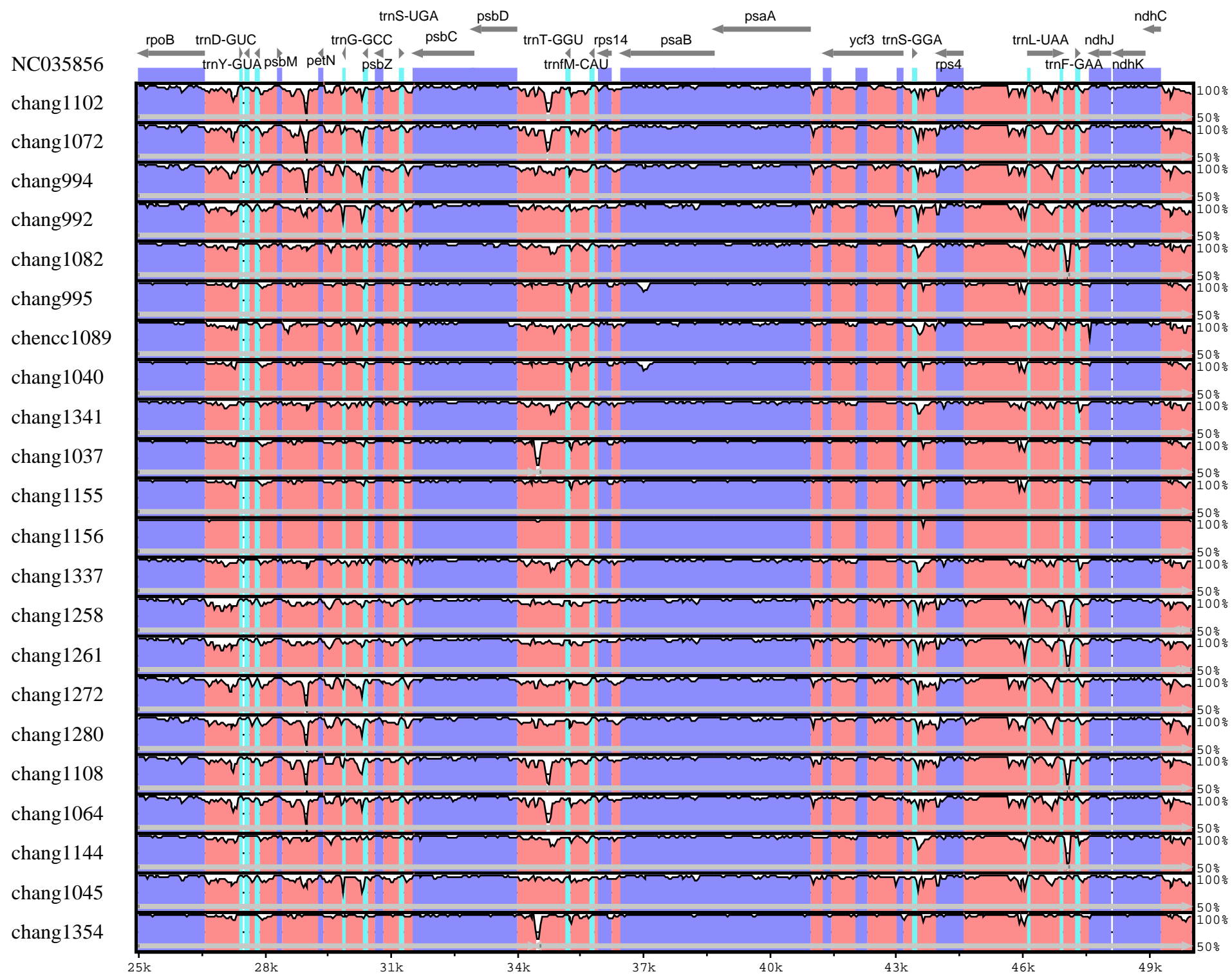

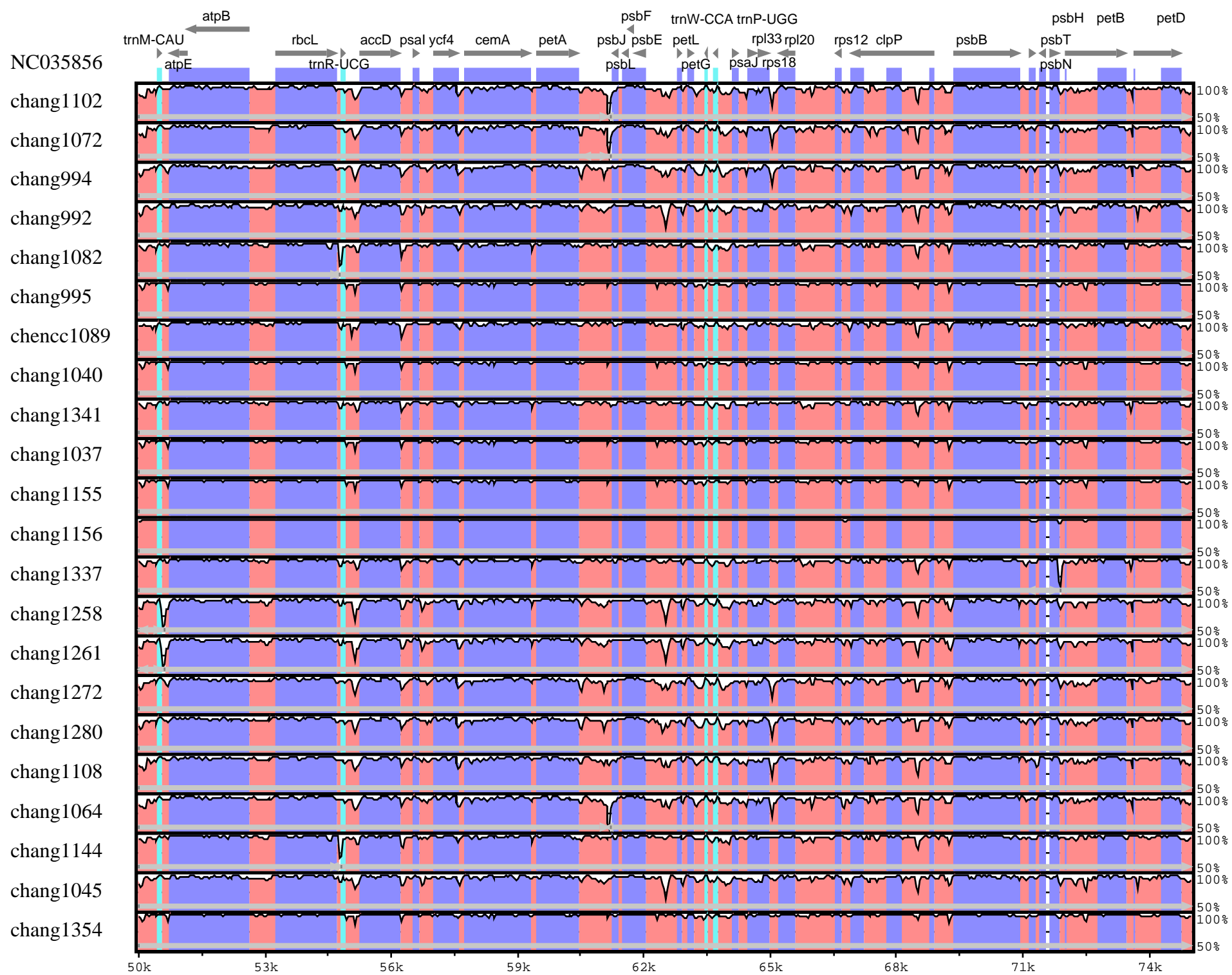

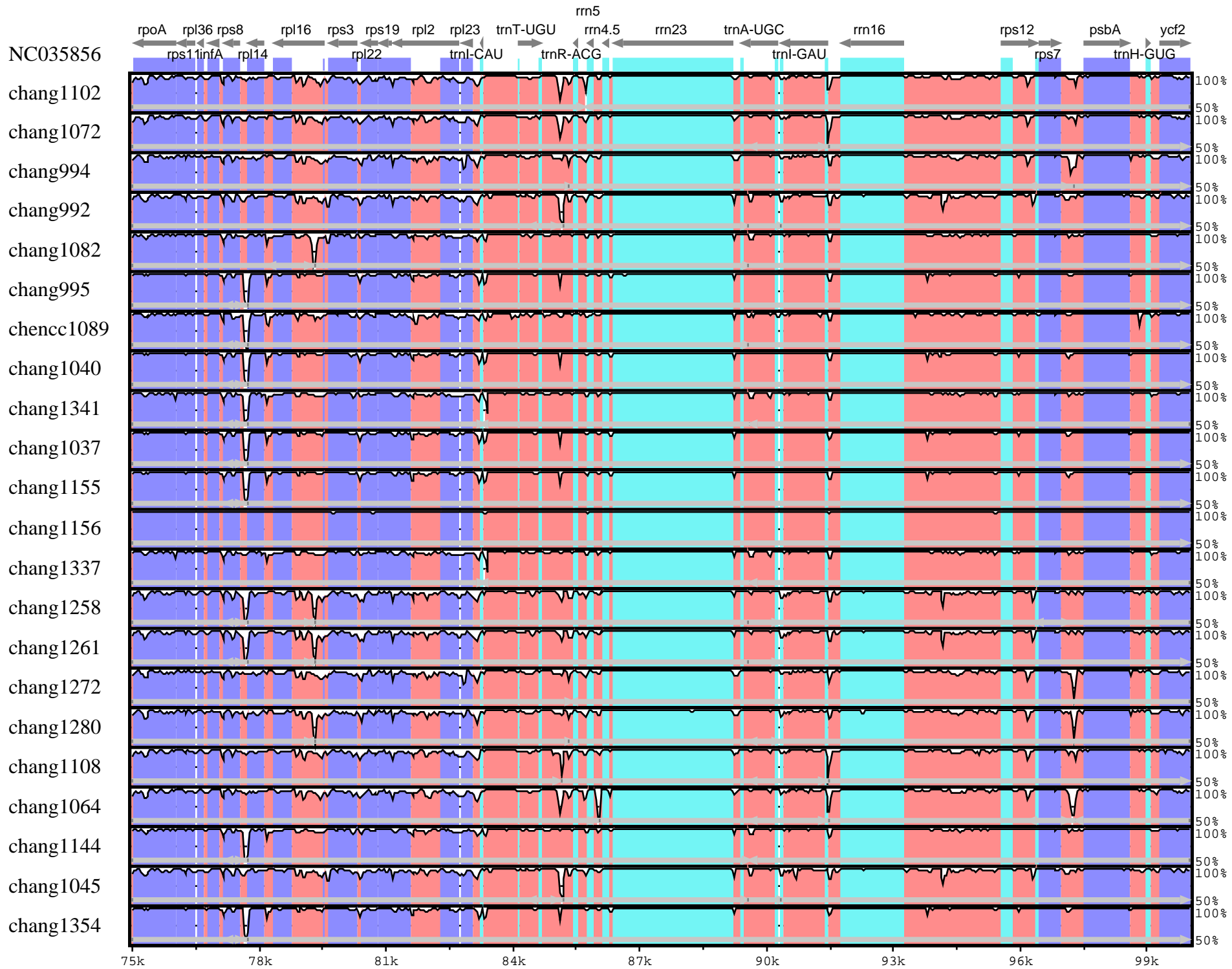

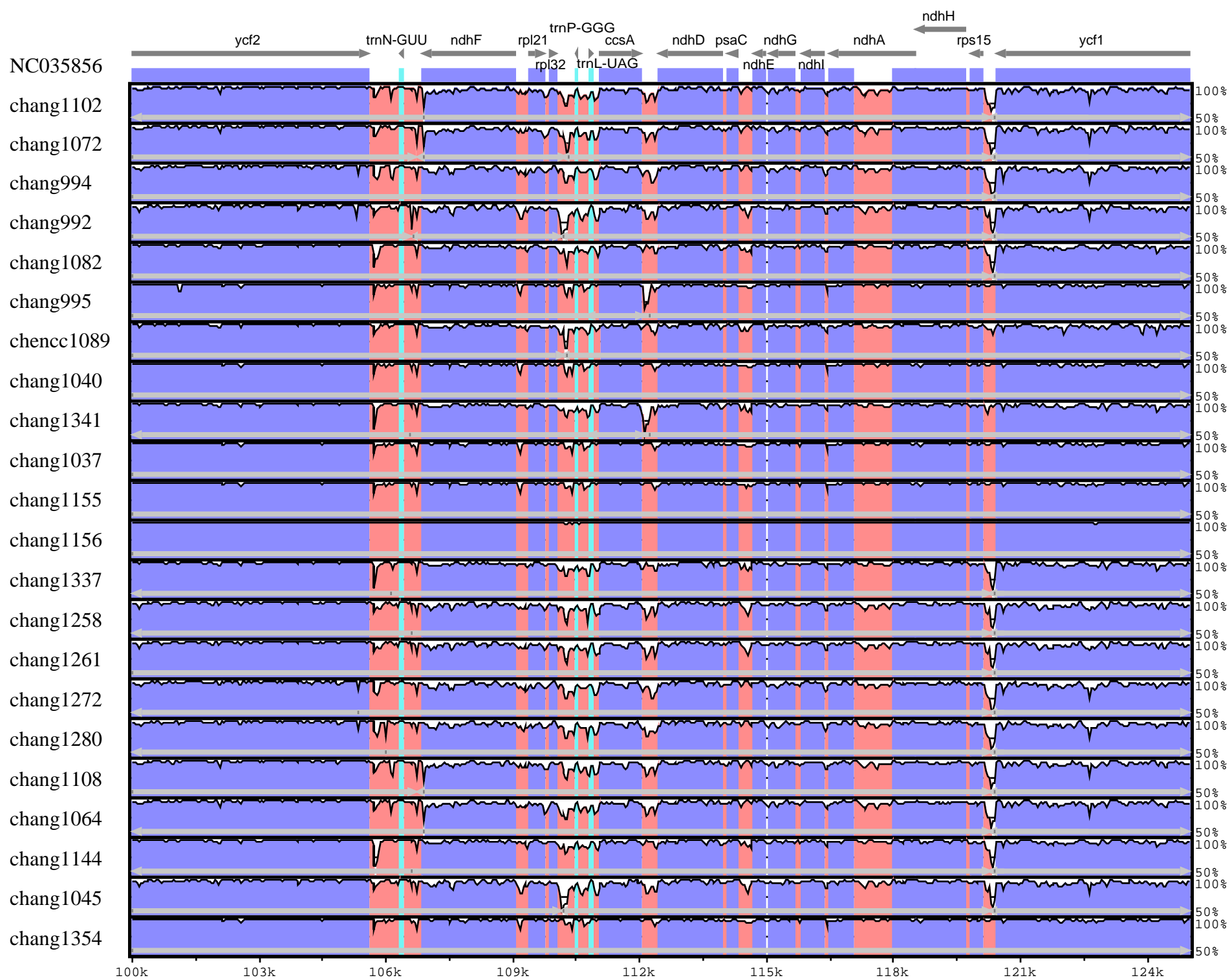

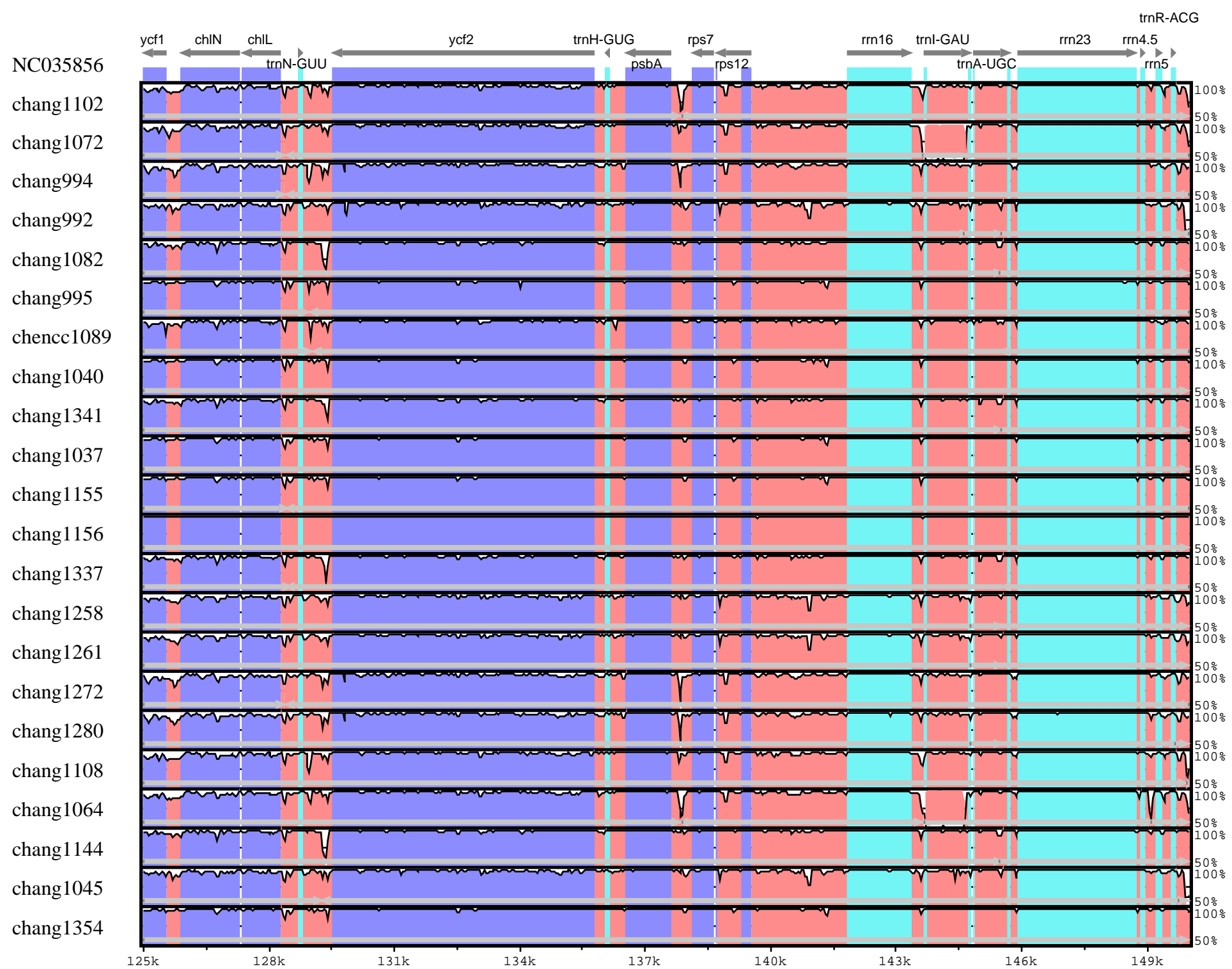

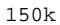

Supplement: Supplemental Information 1 — X-axis: coordinate in the chloroplast genome; Y-axis: level of variation (50%–100%). Genome regions are color-coded, distinguishing between exons (purple), introns (blue), and intergenic-spacers (IGS) (red) [file peerj-12-18667-s001.pdf]
